# Fatty Acid-Induced Lipotoxicity in Pancreatic Beta-Cells During Development of Type 2 Diabetes

**DOI:** 10.3389/fendo.2018.00384

**Published:** 2018-07-16

**Authors:** Yoon S. Oh, Gong D. Bae, Dong J. Baek, Eun-Young Park, Hee-Sook Jun

**Affiliations:** ^1^Department of Food and Nutrition, Eulji University, Seongnam, South Korea; ^2^Department of Molecular Medicine, Lee Gil Ya Cancer and Diabetes Institute, Gachon University, Incheon, South Korea; ^3^College of Pharmacy and Natural Medicine Research Institute, Mokpo National University, Jeonnam, South Korea; ^4^Gachon Institute of Pharmaceutical Science, College of Pharmacy, Gachon University, Incheon, South Korea; ^5^Gachon University Gil Medical Center, Gachon Medical and Convergence Institute, Incheon, South Korea

**Keywords:** fatty acid, beta-cell, lipotoxicity, insulin secretion, type 2 diabetes

## Abstract

Type 2 diabetes is caused by chronic insulin resistance and progressive decline in beta-cell function. Optimal beta-cell function and mass is essential for glucose homeostasis and beta-cell impairment leads to the development of diabetes. Elevated levels of circulating fatty acids (FAs) and disturbances in lipid metabolism regulation are associated with obesity, and they are major factors influencing the increase in the incidence of type 2 diabetes. Chronic free FA (FFA) treatment induces insulin resistance and beta-cell dysfunction; therefore, reduction of elevated plasma FFA levels might be an important therapeutic target in obesity and type 2 diabetes. Lipid signals via receptors, and intracellular mechanisms are involved in FFA-induced apoptosis. In this paper, we discuss lipid actions in beta cells, including effects on metabolic pathways and stress responses, to help further understand the molecular mechanisms of lipotoxicity-induced type 2 diabetes.

## Introduction

**Figure 1:**
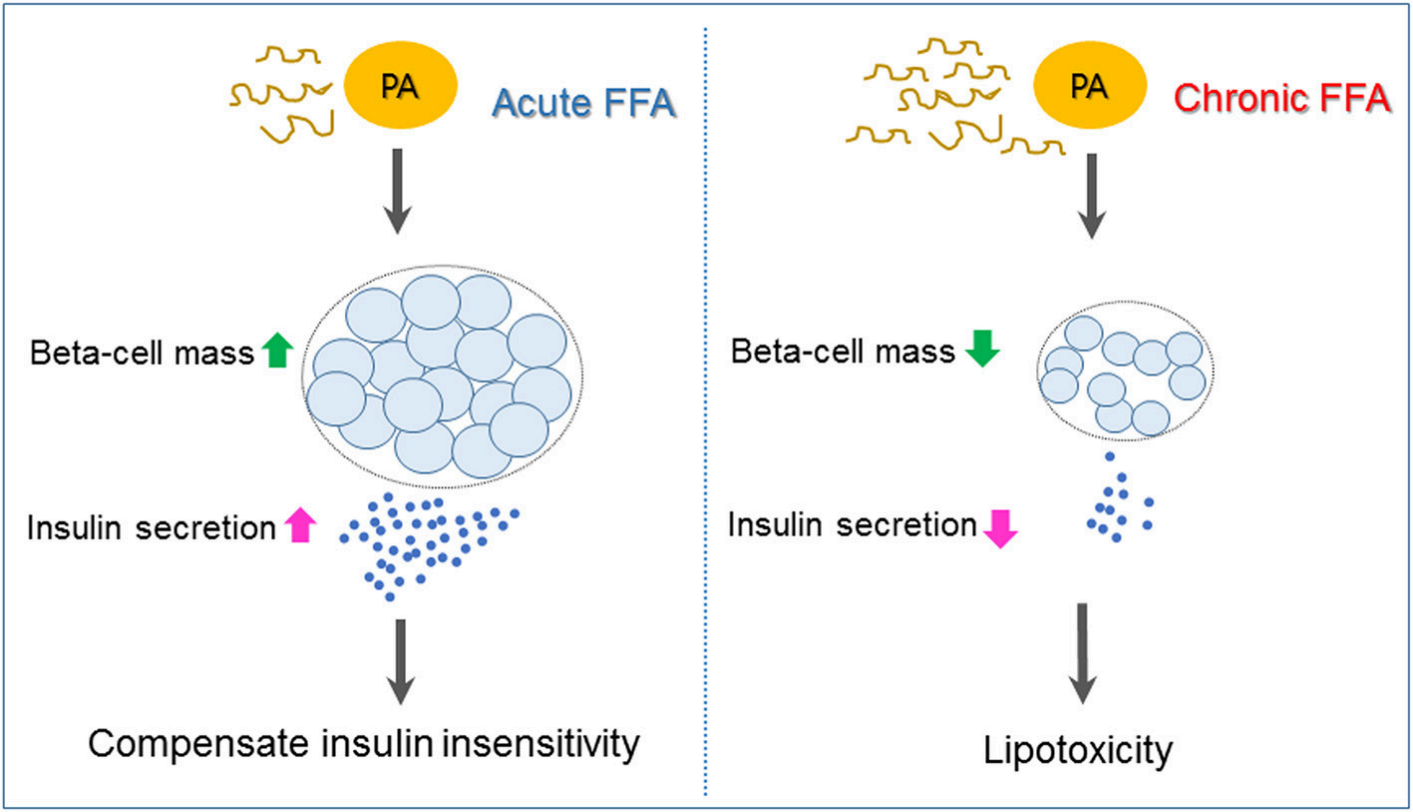
Mechanisms underlying pancreatic beta-cell failure induced by lipotoxicity. When insulin resistance occurs, elevated free fatty acid (FFA) such as palmiate (PA) acutely increases beta-cell mass and insulin secretion to compensate for insulin insensitivity. Chronic increases of plasma FFA result in lipotoxicity, which contributes to beta-cell dysfunction and apoptosis and, as a consequence, induces type 2 diabetes.

Type 2 diabetes is a heterogeneous syndrome that is related to both defective insulin secretion and peripheral insulin resistance. Beta cells are the major organs for secreting insulin; hence, it is important to maintain an adequate beta-cell mass in response to various changes. Free fatty acids (FFAs) are nutrients involved in the energy metabolism of most organisms and are known to regulate beta-cell equilibrium. There is some evidence that elevated fasting and postprandial FFA concentrations increase the risk of developing type 2 diabetes and obesity (1, 2). When insulin resistance occurs, elevated FFA levels acutely increase beta-cell mass and insulin secretion to compensate for insulin insensitivity. However, chronic increases of plasma FFA concentrations result in disturbances in lipid metabolism regulation, which contribute to decreased beta-cell function and viability (lipotoxicity), and consequently induce type 2 diabetes (3) (Figure 1).

Prospective studies with subjects at risk for type 2 diabetes demonstrate that development of abdominal obesity is correlated with loss of beta-cell function (4). Lipotoxicity caused by chronic dyslipidemia impairs the metabolism of lipids in a detrimental cycle, leading to further beta-cell damage (5). In PROMISE cohort study during 6 years, total FFA concentration, but not specific fatty acid composition, was a strong predictor of beta- cell function (6). However, FA composition is one of the critical factors in the induction of lipotoxicity in beta-cells and type 2 diabetes (7).

Fatty acids are classified by carbon chain length. Short-chain fatty acids (SCFA) contain fewer than 6 carbons, medium-chain fatty acids (MCFAs) have 6–12 carbons, and long-chain fatty acids (LCFAs) contain more than 12 carbons. Moreover, according to the double bond configuration, saturated fatty acids (SFA) and unsaturated fatty acids are categorized, that can be classified as mono (MUFA) or polyunsaturated (PUFA) (8). The effects of a specific fatty acid (FA) on insulin secretion and beta-cell survival are related to the degree of saturation and carbon chain length of the FA. Saturated molecules with a chain length of carbon (C)16 or greater [palmitate (C16:0) or stearate (C18:0)] induced cytotoxicity, whereas a reduction of the carbon chain length to C14(C) (myristate) or C12:0 (laurate) are less toxic to beta-cells (9). But unsaturated fatty acids (both mono and poly unsaturated fatty acids) do not induce beta-cell apoptosis and this effects was chain length independent. Treatment of beta cells with an unsaturated FA, such as arachidonic acid (C20:4), increases glucose-stimulated insulin secretion (GSIS) and beta-cell proliferation (10). Docosahexaenoic acid (DHA, C22:6) and eicosapentaenoic acid (EPA, C20:5) also prevent cytokine induced cell death in pancreatic islets and enhance insulin secretion (11). Other studies demonstrated that cytokine treatment induced cell death in the wild type islets, but islets from the mfat-1 transgenic mice (containing DHA and EPA) showed resistance to cytokine induced cell death (12, 13). Therefore, controlled supplementation of PUFAs has been shown to decrease triglyceride and cholesterol levels and enhance insulin secretion (14).

Dietary fats, specifically unsaturated fatty acids can modulate type 2 diabetes development and among them, palmitic acid is one of the main fatty acid involved in the lipotoxicity during the type 2 diabetes progression. Prolonged exposure to palmitate (PA), an ester of the saturated palmitic acid, inhibits the secretory capacity of beta cells, impairs insulin gene expression, and increases beta-cell apoptosis (15).

It is widely reported that lipotoxicity induced by PA promotes beta-cell apoptosis, but the mechanisms are not fully described and many proposed models are still being investigated (14). This review discusses mechanisms, such as expression of receptors, synthesis of *de novo* ceramide, lipid droplet (LD) formation, endoplasmic reticulum (ER) stress, mitochondrial dysfunction, and autophagy, that regulate beta-cell death and dysfunction with a focus on development of type 2 diabetes.

## Fatty acids and lipotoxicity

**Figure 2:**
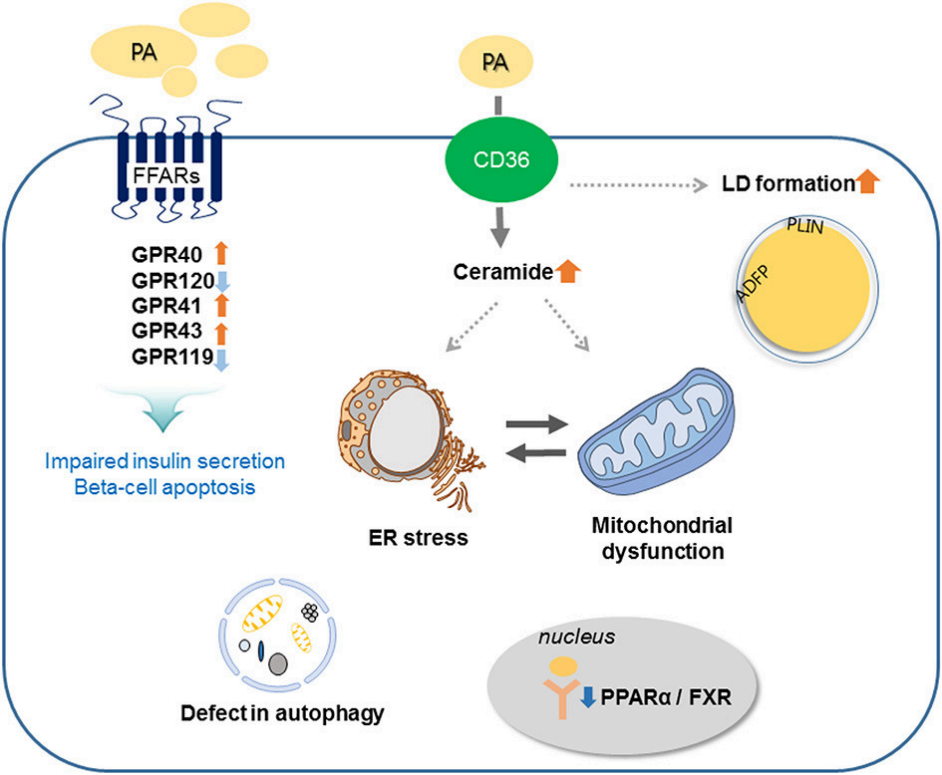
Involved mechanisms regarding impaired insulin secretion and beta-cell apoptosis under lipotoxic condition in pancreatic beta-cells. Palmitate (PA) activates CD36 or FFA receptors (FFARs) and cell stress responses including ceramide formation, lipid droplet (LD) formation, endoplasmic reticulum (ER) stress, mitochondrial dysfunction, and autophagy. These responses result in beta-cell damage and impaired insulin secretion.

Prolonged exposure of isolated islets or insulin-secreting cells to elevated FA levels is associated with inhibition of GSIS, reduction of insulin gene expression, and induction of cell death by apoptosis. Compared to untreated rat islets, rat islets cultured for 7 days in the presence of high levels of FFAs exhibit the hallmark events of apoptosis such as DNA fragmentation, increased caspase activity, ceramide formation, and expression of apoptotic genes (16). When a high-fat diet (HFD) is administrated to non-obese Goto-Kakizaki (GK) rats, beta-cell dysfunction is increased (17). Moreover, intralipid-induced impairment in beta-cell function is accelerated in obese subjects with glucose intolerance and mild hyperglycemia (18). Lipid or FFA exposure activates FFA receptors and cell stress responses including ceramide formation, LD formation, ER stress, mitochondria dysfunction, and autophagy, and these responses result in beta-cell damage and impaired insulin secretion (Figure 2).

### FFA receptors

#### CD36

CD36 is an N-linked glycosylated transmembrane protein that is also known as FA translocase (FAT). After cross the cell membrane via CD36, fatty acids are activated by fatty acyl-CoA synthetase to generate acyl-CoA which undergoes β-oxidation. Acyl-CoA also enters the glycerolipid/free fatty acid cycle or participates in sphingolipid synthesis to generate metabolites such as ceramides and sphingosine-1 phosphate (19). The binding of long chain FFA to CD36 stimulates the tyrosine phosphorylation of downstream proteins, including proinflammatory response associated with diabetes (20). CD36 is upregulated in response to high glucose in beta-cell, and upregulation of CD36 transporter in beta-cells increases uptake of FA, which are amelioration of the GSIS and impaired oxidative metabolism (21). Sulfo-N-succinimidyl derivatives have been developed as selective inhibitors for CD36, and preincubation with CD36 inhibitor prevents saturated FFA-induced apoptosis via reduced reactive oxygen species (ROS) production (22). In addition to role in FFA transport, CD36 has an important role in signal transduction through activation of non-receptor tyrosine kinases of the Src family (20). These results suggested that CD36 could be a therapeutic target for the treatment of diabetes induced by lipotoxicity.

#### G-protein coupled receptors (GPRs)

FFAs bind to GPRs and regulate insulin secretion pathways. Four FFA receptors, FFAR1 (GPR40), FFAR2 (GPR43), FFAR3 (GPR41), and FFAR4 (GPR120), are expressed in human and rodent beta-cells, but those receptors have different chain length specificities, and the degree of saturation affects insulin secretory function (23). FFAR1 and FFAR4 are activated by medium- and long-chain FAs, while the other two receptors are activated by short-chain FAs. Among the receptors, FFAR1 and FFAR4 are the most closely related to lipotoxicity-induced beta-cell apoptosis.

FFAR1 (GPR40) is activated by medium- and long-chain FFAs [especially, ecosatrienoic acid (C20:3)] and facilitates GSIS in pancreatic beta cells (24, 25). Insulin secretory effect of FFAs on beta-cells was decreased by loss of FFAR1 function. Steneberg et al. demonstrated that loss of FFAR1 protects mice from obesity induced hyperinsulinemia, hyperglycemia and glucose intolerance, but overexpression of FFAR1 in beta-cell of mice leads to impaired beta-cell function and diabetes (26). PA treatment of human islets decreases insulin content and secretion, and those decreases can be prevented by treatment with FFAR1 antagonists (27). These results suggested that FFAR1 antagonists may have therapeutic benefits. However, other studies showed that upregulation of FFAR1 protects against lipotoxicity in rat insulinoma (INS-1) cells (28), while FFAR1 overexpression in islet beta cells improves GSIS and glucose tolerance *in vivo* (29). In human study, a single nucleotide polymorphism at the FFAR1 locus is correlated with insulin secretory dysfunction (30). FFAR1 is also expressed intestinal L and K cells, which secrete incretin hormones such as glucagon-like peptide 1 (GLP-1) and glucose-dependent insulinotropic polypeptide (GIP) (31), suggested that FFAR1 regulate FFA-induced insulin secretion from beta-cells directly and indirectly by regulation of incretin secretion. Therefore, many pharmaceutical companies and academic institutes are undertaking development of FFAR1 agonists such as Tak-875, LY2881835, and AMG-837 (32). Tak-875 reduced glycemia in diabetic patients but not in normoglycemic people without diabetes and progressed to phase III clinical trials (33). However, the development was discontinued because of hepatotoxicity (34). Recently, some agonist such as P11187, LY2922470, and SHR0534 are currently in phase I clinical trials (32).

FFAR4 (GPR120) is unsaturated FFA (ω-3, ω-6, and ω-9) and saturated FFAs with long- chain carbon sensor. Among them, two ω-3 FAs, docosahexaenoic acid (DHA) and α-linoleic acid, are the most potent and most common GPR120 agonists (35). GPR120 increases insulin release from beta cells via increases in incretin (glucagon-like peptide-1) secretion by intestinal cells (35) and exhibits an anti-apoptotic effect via activation of extracellular signal-regulated kinase (ERK) and phosphoinositide 3-kinase (PI3K)-Akt (protein kinase B) (36). FFAR4 KO mice develop glucose intolerance, and a dysfunctional variant of FFAR4 (R279H) is associated with obesity in humans (37). Therefore, activation of pancreatic GPR120 may mediate the anti-apoptotic effects of poly unsaturated FAs in diabetic beta cells.

FFAR2 (GPR43) and FFAR3 (GPR41) are short-chain FA [propionate (C3), butyrate (C4), and valerate (C5)] receptors that mediate inhibition of insulin secretion by coupling with G proteins. Genetic deletion of both receptors in beta- cells show enhanced insulin secretion and improved glucose tolerance in HFD-fed diabetic mice compared with that in controls (38). However, GPR43 and GPR41 are involved in GLP-1 secretin from intestinal L cells. They were abundantly expressed in L cells and knockout mice of these receptors exhibited reduced short chain FA-mediated GLP-1 secretion both *in vitro* and *in vivo* and results in impaired glucose tolerance (39). These results suggest that development of agonist or antagonists of GPR43 and GPR41 may be expected to be efficacious in improving insulin secretion in type 2 diabetic subjects.

Agonists for GPR119, a highly expressed beta-cell receptor for FA metabolites (lysophosphatidylcholine, oleoylethanolamine), act as insulin secretagogues. A GPR119-specific agonist, AR231453, increases insulin release in HIT-T15 cells (a hamster pancreatic beta-cell line) and rodent islets. Moreover, AR231453 treatment in diabetic KK-Ay mice improves glucose tolerance (40). N-oleoyldopamine, a lipid amide, activates GPR119, enhances insulin secretion in RIN-5F cells (a rat islet cell line), and improves glucose tolerance when administered orally to C57BL/6J mice (41).

#### Nuclear receptors

Peroxisome proliferator-activated receptor alpha (PPAR-α) serves as a long-chain FA sensor and regulates FA metabolism by decreasing lipid content and minimizing lipotoxicity (42). PPAR-α has a role in protecting beta-cells from oleate-induced dysfunction. In INS-1 cells, insulin secretory dysfunction induced by oleate is accelerated by PPAR-α siRNA treatment, and overexpression of PPAR-α stimulates insulin secretion in human islets (43).

Farnesoid X receptor (FXR), a bile acid receptor, is another key nuclear receptor regulating beta-cell function in human islet and beta-cell cell lines (44). FXR is predominantly localized in the cytosol of islets of normal mice but translocates to the nucleus under a diabetic condition. GSIS is impaired in islets of FXR KO mice, and FXR activation protects human islets from lipotoxicity and enhances insulin secretory function (44). V-maf musculoaponeurotic fibrosarcoma oncogene homolog (Maf) A, Beta2/Neuro D1, and pancreatic and duodenal homeobox (Pdx)-1 (a master transcription factor regulating the insulin gene), and GSIS are reduced in FXR KO islets. The molecular mechanisms by which FXR activation leads to insulin secretion and protects lipotoxicity is unclear, but expression of FGF-19, a member of the FGF family was increased by treatment with FXR agonist and increased FGF-19 might be reduced palmitate induced triglyceride accumulation and apoptosis (44).

### Ceramide formation

Exposure to excess long-chain saturated FFAs [palmitate (16:0), stearate (C18:0), arachidate (C20:0)] and linocerate (C24:0), but not shorter saturated [myristate (C12:0)] or unsaturated FFAs induces ceramide accumulation by serine palmitoyl transferase and ceramide synthase (CerS) (45, 46). *de novo* ceramide synthesis has been suggested to be a mediator of FFA-induced beta-cell toxicity. Overexpression of CerS4 potentiates PA-induced accumulation of ceramides and enhances apoptosis through the production of additional toxic ceramide species such as C18:0, C22:0, and C24:1 (47). The C2-ceramide, an analog of ceramide, is able to potentiate the pro-apoptosis and anti-proliferative effects of PA in beta-cells (48). ER stress, alterations in mitochondrial membrane integrity, and inhibition of Akt by ceramide are proposed to induce apoptosis in beta-cells. Synthetic ceramide is accumulated in the ER of beta cells and reduces ER sphingomyelin (SM) and cholesterol, which results in the disruption of ER lipid rafts (49). Lei et al. demonstrated that inhibition of SMase, an enzyme that hydrolyzed SM to generate ceramide, protects beta-cells from ER stress-induced apoptosis (50). Also, ceramide increases mitochondrial membrane permeability and leads to the activation of intrinsic pathways via decreased anti-apoptotic molecules Bcl-2 and increased caspase 3/7 (47). Ceramide has been shown to disrupt electron transport at complex I and complex III, resulting in enhanced ROS generation, which facilitates cytochrome c release and caspase activation (51). Akt inactivation by ceramide is involved in the mechanisms by which ceramide causes beta-cell apoptosis. Inhibition of ceramide biosynthesis restores Akt activation (52), and Akt inactivation with ceramide accumulation is observed in human treated with saturated fat (53).

Inhibition of *de novo* ceramide synthesis by using serine palmitoyl transferase (L-cycloserine) or ceramide synthase (fumonisin-B1) inhibitors attenuates FFA-induced beta-cell apoptosis and lowers hyperglycemia (54). Tang et al. reported that beta-cell-secreted active neutral ceramidase protects beta cells from FFA-induced apoptosis through regulation of sphingolipid metabolites (55).

As ceramide and its derivatives have a variety of roles in beta-cell biology, further investigation will help to elucidate the mechanisms underlying beta-cell failure caused by lipotoxicity.

### Lipid droplet formation

Lipid droplets (LDs) is one of the important organelles in cellular energy balance. LD contains a core or neutral lipid (triglyceride (TG) and cholesterol ester) coated by an interface composed of a monolayer of phospholipids, free cholesterol and proteins (56). The storage droplets help transport the neutral lipids to specific cellular destinations or direct them to specific metabolic pathway. Such metabolic pathway was controlled by the LD coat proteins of the perilipin family such as perilipin (PLIN), adipocyte differentiation-related protein (ADFP), tail-interacting protein of 47 kilodaltons (TIP47) and oxidative tissue-enriched PAT protein (OXPAT) (57). PLIN protects against lipotoxicity when overexpressed beta-cells (58), but recently reported that that downregulation of PLIN2 ameliorates chemical induced ER stress (59). Identifying the role of PLIN on the lipotoxic beta-cells will be elucidated in the future. Besides PLIN, ADFP also plays a crucial function in intracellular lipid metabolism. Expression level of ADFP was increased in mouse islets from HFD administration and downregulation of ADFP in beta-cells results in the suppression of TG accumulation upon FA loading (60). However, few studies focused on the association of ADFP and beta-cell function under lipid stress. Further studies of PLIN and ADFP to increase understanding of lipid droplet formation in lipotoxic beta-cells will be needed.

### ER stress

The ER is one of the important metabolic organelles playing a key role in beta-cell function. Activation of ER plays a crucial role in the synthesis, correct folding and sorting of insulin in response to glucose. ER forms the main intracellular Ca^2+^ reservoir and the controlled release of Ca^2+^ into the cytosol is a critical step for insulin synthesis. Therefore, beta-cells are particularly sensitive to ER stress and unfolded protein response (UPR) such as ER transmembrane proteins PKR-like endoplasmic reticulum kinase (PERK), inositol-requiring enzyme (IRE)-1, and activating transcription factor (ATF)-6. Saturated FAs promote ER stress and induce beta-cell apoptosis (61, 62). ER stress markers are elevated in pancreatic islets in animal models of diabetes and in patients with type 2 diabetes (63). Chemical chaperone 4-phenylbuturic acid treatment restores ER morphological changes induced by PA (64), and deletion of C/EBP homologous protein (CHOP, transcription factor in the ER stress response) in HFD-fed mice improves beta-cell function and promotes cell survival (65).

Many pathways were involved in the regulation of ER stress induced apoptosis. Activated UPR pathways have been directly linked to the intrinsic apoptotic pathway (62). Proapoptotic signals of c-Jun N-terminal kinase (JNK), induced by PA, are activated downstream of IRE-1. Eukaryotic translation initiation factor 2 alpha subunit (eIF-2α) phosphorylation via PERK leads to loss of the myeloid cell leukemia sequence 1 (MCL1) protein, which is an anti-apoptotic member of the BH3 family (66). In addition, PA-induced ER stress interacted with the inflammatory response by activating several proinflammatory pathway, such as NF-kB, JNK, double stranded RNA-dependent protein kinase and nucleotide-binding oligomerization domain NLRP inflammasome (67).

Disruption of protein processing and trafficking or incorrect Ca^2+^ regulation in ER are involved in FA-induced beta-cell apoptosis. Compared with cytokines and glucotoxicity, PA efficiently decreased ER Ca^2+^ levels (68), and reduced Ca^2+^ levels in ER triggers the unfolded protein response to rescue cells from misfolded protein overload or programmed cell death (68). Marnugi et al. reported that Sorcin, a calcium sensor protein in ER, is downregulated under lipotoxic stress conditions, and resulting in ER stress and beta-cell dysfunction (69). Santulli et al. demonstrated that mutation of type 2 ryanodine receptor (RyR2), Ca^2+^ release channel on the ER, caused activated ER stress response, mitochondrial dysfunction and results in impaired insulin secretion and glucose homeostasis (70). The accumulation of misfolded protein causes ROS generation from the oxidative folding process in the ER and mitochondria. Defective disulfide bond formation reduces glutathione in the ER and produces oxygen radicals (71). Lipotoxicity also disrupts ER-to-Golgi protein trafficking, resulting in impaired proinsulin maturation and loss of insulin content (72).

The UPR causes accumulation of human islet amyloid polypeptide, which occurs in 90% of type 2 diabetic patients (73). Many studies have demonstrated that islet amyloid polypeptide formation is cytotoxic (74, 75) because amyloid polypeptide accumulates intracellular ROS and induces lipid peroxidation (74).

There are close connections between oxidative stress and organellar Ca^2+^ homeostasis. Interaction between ER and mitochondria was involved in the lipotoxicity- induced ER stress via regulation of Ca^2+^ signaling (76). Prolonged ER stress leads to release of Ca^2+^ from the ER lumen at the mitochondria-associated membranes (NAM) and consequently leads to increased Ca^2+^ uptake into the mitochondrial matrix. Prolonged mitochondrial Ca^2+^ accumulation triggers opening of the mitochondrial permeability transition pore (mtPTP) and these results in swelling the organelle, rupture of the outer mitochondrial membrane and release of proapoptotic protein into the cytosol (77). Ly et al. also suggested that palmitate induced deprivation of Ca^2+^ from ER and this leads to ER stress and CHOP upregulation. Moreover, released Ca^2+^ transfers into mitochondria and mitochondrial Ca^2+^ overload causes superoxide production and induces apoptosis (78).

These results suggest that several mechanisms are involved in lipotoxicity-induced ER stress. Therefore, reducing ER stress in beta- cells could lead to novel and efficient therapeutic treatments for palmitate-induced lipotoxicity. Several studies have demonstrated that knockdown of ER stress proteins (ex, CHOP) has protective effects on palmitate-induced apoptosis in beta cells (79), and chaperones such as taurine-conjugated ursodeoxycholic acid (TUDCA) and 4-phenylbutyruc acids have been tested to protect from PA-induced ER stress and apoptosis (80).

### Mitochondrial dysfunction

Mitochondria play an essential role in adenosine triphosphate (ATP) synthesis, Ca^2+^ homeostasis, and the integration of apoptotic signals (81). In beta-cells, glucose sensing and subsequent insulin secretion was controlled by mitochondrial metabolism. During the glycolysis and tricarboxylic acid (TCA) cycle, reduced form of nicotinamide adenine dinucleotide (NADH) or flavin adenine dinucleotide (FADH2) are generated and electron transfer to the mitochondrial electron-transport chain (ETC) leads to production of ATP via the process of oxidative phosphorylation. Increased in ATP/ADP ratio allows Ca^2+^ uptake, contributing to secretion of insulin (82). Therefore, defects in mitochondrial function impair this metabolic process and consequently promote apoptosis and beta-cell death. Various factors have been identified that may contribute to mitochondrial dysfunction.

Increased FA levels leads to incomplete FA oxidation and induces ROS production, with concomitant mitochondrial stress, which leads to lipotoxicity (83). PA is known as a potent inducer of ROS and ROS attack insulin secreting cells and these results in mitochondrial inactivation and interruption of signal transduction correlated with insulin secretion (84). CD 36 is required for FA-induced ROS production and proinflammatory pathways (7).

Mitochondrial electron transport chain is an important site of ROS production within the cells, but beta-cells are low in antioxidant enzymes such as catalase, glutathione peroxidase, and superoxide dismutase, thus they are sensitive to ROS (85). Human islets from diabetic individuals show lipid peroxide protein adducts (86) and lipid infusion increases islet ROS and impairs insulin secretion (87). These lipid peroxides have lipotoxic effects on mtDNA, RNA and proteins of the mitochondrial machinery, leading to mitochondrial dysfunction (88).

Mitochondrial uncoupling refers to the dissociation of electron-dependent oxygen consumption from ATP generation. Uncoupling protein (UCP)-1,−2, and−3 are expressed in a tissue-specific manner, but only UCP-2 is expressed in pancreatic beta cells (89). Increased expression of UCP-2 is observed in islets of HFD-fed rodents and in FFA-treated islets (90), and activation of UCP-2 attenuates GSIS. Islets from UCP-2 KO mice show resistant to PA-induced cellular toxicity and remains normal insulin secretion (91). However, another report demonstrated that UCP-2 KO in mice causes oxidative stress and impairs GSIS (92). Recently, it was reported that UCP-2 is not involved in PA-induced impairment of insulin secretion in INS-1 cells (93). As there are contrasting results on the biochemical and physiological functions of UCP-2, further clarification of the role of UCP-2 in the lipotoxicity of beta-cells and the pathogenesis of diabetes is needed.

Mitochondrial morphology contributes to the maintenance of insulin levels by regulating apoptosis and beta-cell mass. Exposure of beta-cells to glucolipotoxicity induces mitochondrial fragmentation and restoring normal morphology prevents apoptosis (94). Mitochondria dynamics are modified by fission and fusion, as fusion can compensate for damage to the contents of dysfunctional organelles by fusing them with functionally competent ones, whereas fission drives damaged organelles to mitophagy and prevent apoptosis (95). It was reported that beta-cell specific deletion of autophagy related 7 (Atg-7) results in dispersed, small, and swollen mitochondria and accompanied by reduced beta-cell mass with reduced GSIS (96). It was reported that disconnected, swollen and shorter beta-cell mitochondria was observed in Zucker diabetic fatty rats (ZDF) (97) and beta-cells from diabetic patients (98). Molina et al. reported that PA treatment results in mitochondrial fragmentation and impairs network dynamics; moreover, manipulations that shift to fusion prevent lipotoxicity-induced apoptosis (99). In addition, Wiederkehr et al. demonstrated that inhibition of mitochondrial networking augments sensitivity to lipotoxicity (100). These results indicated that mitochondrial morphodynamics such as fusion and fission are involved in lipotoxicity in beta-cells and in the pathophysiology of type 2 diabetes.

Further elucidation of mitochondrial dysfunction and identification of mitochondrial targets against lipotoxicity will be helpful in identifying pharmacological targets for the protection of beta-cell mass and beta-cell function in type 2 diabetic subjects.

### Autophagy

Autophagy is a dynamic process that has a major role in the elimination of pathogens, dysfunctional organelles, and protein aggregates through lysosomal mechanisms. Upon induction of stress such as ROS exposure or ER stress, autophagy is stimulated to protect the cell by clearing accumulated damaged components. The activating complex UNC-51-like kinase (ULK1)/ autophagy-related protein (ATG)1, the Beclin/PI3K (VPS34) complex, two transmembrane proteins (ATG9 and VMPL), two ubiquitin-like conjugation systems (ATG12/ATG5 and ATG8/LC3) are molecular components involved in the autophagy process (101). Under normal condition, autophagy is inhibited by the activation of the mTORC1 complex, a modulator activated during the insulin pathway or in states of abundant nutrients. However, during energy reduction or in the presence of mTOR inhibitors, ATG proteins recruited to form a autophagy complex (102).

The role of autophagy in FFA-induced toxicity is unclear. When appropriate stimulation occurs, autophagy is activated as a survival mechanism (103). Numbers of autophagosomes are high in ZDF rats, db/db mice, and HFD-fed C57BL/6 mice (104). Long term treatment with PA or oleate to INS-1 cells show increased autophagosome numbers, and activation of autophagosomes indicates their protective role against PA-induced death (105, 106). Mice with a beta-cell-specific KO of ATG7 display impaired glucose tolerance as well as impaired insulin secretion after being fed a HFD (105), and reduction of autophagosome formation augments PA-induced beta-cell death (107). Choi et al also demonstrated that ATG5 downregulation enhanced susceptibility to cell death induced by lipotoxicity but stimulation of autophagy using rapamycin ameliorated lipotoxicity (106). These results suggested that increase autophagy in response to FA plays a protective mechanism from lipotoxicity.

In contrast, activation of autophagy by FAs in beta-cells has been reported to be an apoptotic signal. ATG7 overexpression sensitizes cells to PA-induced autophagy, which increases inflammatory mediators via cathepsin B and NLRP3 inflammasome, resulting in exacerbation of lipotoxicity in INS-1 cells (108). Ebato et al. also reported that genetic deletion of ATG7 in beta-cells results in degeneration of islets and impaired glucose tolerance with reduced insulin secretion during high fat diet (105).

These contrary results suggest that induction of autophagy has either detrimental or protective roles in beta cells; therefore, the role of autophagy in beta-cell failure in type 2 diabetes requires further investigation.

## Conclusion

Beta-cell failure is a major risk factor at the onset and during progression of type 2 diabetes. FFAs have both positive and negative effects on beta-cell survival and insulin secretory functions. However, chronic PA treatment results in lipotoxicity and beta-cell dysfunction, consequently resulting in type 2 diabetes. Several FFARs that are specifically activated by FFAs, disturbances in lipid metabolism and intracellular pathways, including cellular stress responses such as oxidative stress, ER stress, autophagy, and ceramide/LD formation are involved in lipotoxicity-induced beta-cell death. This review helps to understand the molecular mechanisms of lipotoxicity-induced type 2 diabetes, and identification of the molecular mechanisms related to FFAs that regulate beta-cell mass and function could provide guidance in the development of new therapeutic targets for diabetes.

## Author contributions

E-YP, DB, and GB collected information. YO and H-SJ collected information and wrote the manuscript.

### Conflict of interest statement

The authors declare that the research was conducted in the absence of any commercial or financial relationships that could be construed as a potential conflict of interest.
